# National Survey Indicates that Individual Vaccination Decisions Respond Positively to Community Vaccination Rates

**DOI:** 10.1371/journal.pone.0166858

**Published:** 2016-11-21

**Authors:** John Romley, Prodyumna Goutam, Neeraj Sood

**Affiliations:** 1 Price School of Public Policy, University of Southern California, Los Angeles, California, United States of America; 2 School of Pharmacy, University of Southern California, Los Angeles, California, United States of America; 3 Leonard D. Schaeffer Center for Health Policy and Economics, University of Southern California, Los Angeles, California, United States of America; 4 Pardee RAND Graduate School, RAND Corporation, Santa Monica, California, United States of America; University of Waterloo, CANADA

## Abstract

Some models of vaccination behavior imply that an individual’s willingness to vaccinate could be negatively correlated with the vaccination rate in her community. The rationale is that a higher community vaccination rate reduces the risk of contracting the vaccine-preventable disease and thus reduces the individual’s incentive to vaccinate. At the same time, as for many health-related behaviors, individuals may want to conform to the vaccination behavior of peers, counteracting a reduced incentive to vaccinate due to herd immunity. Currently there is limited empirical evidence on how individual vaccination decisions respond to the vaccination decisions of peers. In the fall of 2014, we used a rapid survey technology to ask a large sample of U.S. adults about their willingness to use a vaccine for Ebola. Respondents expressed a greater inclination to use the vaccine in a hypothetical scenario with a high community vaccination rate. In particular, an increase in the community vaccination rate from 10% to 90% had the same impact on reported utilization as a nearly 50% reduction in out-of-pocket cost. These findings are consistent with a tendency to conform with vaccination among peers, and suggest that policies promoting vaccination could be more effective than has been recognized.

## Introduction

The Advisory Committee on Immunization Practice at the U.S. Centers for Disease Control and Prevention currently recommends vaccines for the prevention of seventeen diseases that afflict individuals of all ages.[1, 2] Health People 2020, a federal initiative, announced an ambitious and comprehensive set of objectives for increasing immunization rates and thereby reducing preventable infectious diseases.[3]

Achieving such objectives remains a major public health challenge in the U.S. and elsewhere.[4] For example, the percentage of California kindergarten children meeting the vaccination requirement for measles, mumps and rubella has declined from 95.5% in 2004 to 92.6% in 2014, notwithstanding the high efficacy of immunization in preventing infection with these sometimes fatal diseases.[5]

Vaccination rates are much higher in some places than in others. During the 2013–2014 flu season, while slightly more than half of elderly Medicare beneficiaries in the state of Florida received a flu shot, the rate ranged from 27% in one county to 62% in another.[6] These stark differences raise important questions about the determinants of vaccination behavior.

Some models of vaccination behavior imply that an individual’s willingness to vaccinate could be negatively correlated with the vaccination rates of peers in the community.[7, 8] The rationale is that a higher community vaccination rate reduces the risk of contracting the vaccine-preventable disease and thus reduces the individual’s benefit from vaccination. This notion that individuals tend to “free ride” on the vaccination decisions of their peers has been used as a justification for government interventions to increase vaccination rates.[9]

At the same time, individuals may also have a tendency to conform to their peers, and an extensive literature that has found conforming or “positive” peer effects in a variety of health related settings. For example, a famous study by Christakis and Fowler showed that an individual with a friend who became obese was nearly 60% more likely to become obese herself.[10] A similar pattern has manifested itself for smoking cessation.[11]

The direction of any peer effects in vaccination behavior has important implications. If peer effects are positive on balance, interventions that promote vaccination can have a multiplier effect, with the direct effect on particular subjects of the intervention amplified by their peers. In this case, positive peer effects mitigate any reduction in incentives to vaccinate due to the reduced risk of disease resulting from herd immunity. On the other hand, if the overall direction of peer effects is negative, then interventions that increase community vaccination will be less effective as gains in community vaccination will be partially offset by the reduced incentive to vaccinate stemming from reduced disease risk due to herd immunity. In this case, interventions are less effective and/or costlier.[12]

Empirical evidence has begun to emerge concerning the influence of peers on individual decisions about vaccination [13–15], but the literature is not extensive, and has some methodological limitations. This study contributes to our current scientific knowledge of the potential influence of peers on vaccination decisions. To do so, we used a rapid survey technology to ask a large sample of U.S. adults about their willingness to use a hypothetical vaccine for the Ebola disease. A number of such vaccines have been under development, with one having completed a successful trial in west Africa.[16–18]

### Conceptual background

The Health Belief Model posits that health-related behaviors are determined assessments of their benefits and costs.[19, 20] These assessments are grounded in information that can be objective or subjective in nature; information can also be global or local, the latter relating to an individual’s social neighborhood.[7]

In the vaccination context, Funk, Salathé and Jansen (2010) provide a systematic review of theoretical models of human behavior and the epidemiology of infectious disease.[7] There is evidence that vaccination behavior is related to numerous psychological, medical, economic, demographic and social factors.[13, 14, 20–26] For example, in a nationally representative survey, Gu and Sood found that individuals who are at high risk of infection are less likely to take a vaccine.[27]

Philipson has shown that parents tend to vaccinate their children at younger ages in U.S. states in which the prevalence of measles is relatively high, and so the benefit from vaccination is high.[28] Similarly, the benefits of vaccination to an individual are high when the risk of contracting a disease from an infectious community member is high, potentially due to a low rate of vaccination within the individual’s community (clearly, the magnitude of infection risk depends on the force of infection as well as behavior, and is greater for a disease like measles than for a disease like hepatitis B.) Where the community vaccination rate is higher and so (all else equal) the risk of infection is lower, the benefits of vaccination to an individual are lower. Paradigms such as the Health Belief Model suggest that an individual may decide to vaccinate when her community vaccination rate is low, but opt against it when the community rate is high.[7, 8]

However, a negative response to the decisions of others to vaccinate could be counteracted by the positive peer effects seen in other health contexts.[10, 11] An individual could be more likely to vaccinate when the community rate is high, because she wishes to conform to the norms of her community. Moreover, an individual may be more likely to learn from her peers about the benefits of vaccination, when more of her peers have undergone the experience.[15, 29–33] A tendency to conform works against an incentive to free ride, and so the impact of community vaccination rates on individual decision-making is uncertain.

## Methods

### Survey instrument

We developed a survey instrument that assessed a number of issues related to the recent Ebola outbreak, including the willingness of respondents to use a potential vaccine. With respect to the latter, we used the “referendum” format which asks survey respondents to vote (that is, answer with a choice of) yes or no for particular scenarios. This format was recommended -–due to its realism and lack of incentive to misreport—by an expert panel tasked with considering how best to ascertain the societal value of goods (such as environmental preservation) which are not usually bought and sold in the marketplace.[34]

In our setting, respondents were first provided with background information about Ebola (including how it spreads), and then shown attributes of a hypothetical vaccine in terms of treatment efficacy, side effects and mode of administration, with each attribute calibrated to the vaccine which was farthest along in the development pipeline at the time, and has since completed a successful trial in west Africa.[16–18] Respondents were presented with “low” and “high” rates of vaccination in their community, and indicated their willingness to use the hypothetical vaccine in each scenario. Low and high rates were specified as 10% and 90%, to ensure that the range was wide enough that any response to community vaccination would manifest itself. Moreover, these rates span current rates of receipt of recommended vaccinations in the U.S.; for example, 13% of males aged 19–21 years had ever received a single dose of the HPV vaccine as of 2014, while 72% of elderly individuals received an influenza vaccination in the 2013–2014 season, and 92% of children aged 19–35 months were vaccinated for measles, mumps and rubella.[35, 36] Respondents were free to define community as they saw fit. Each respondent was randomized to an out-of-pocket vaccine cost ($25, $100, and $250) common to the two community scenarios. In addition, the survey asked respondents about their degree of concern—“very worried,” “somewhat worried,” “not too worried” and “not at all worried”—that they or their families would get sick from the disease.

The survey instrument is included in S1 Appendix Section D. The instrument was screened for comprehensibility by testers from the Understanding America Study (UAS), which fielded the survey and is described below. In addition, survey participants were given the opportunity to provide open-ended feedback, and early responses were reviewed for concerns about clarity and validity. The study was approved by the University Park Institutional Review Board at the University of Southern California.

### Survey administration

The survey was put into the field from October 28, 2014, and we obtained responses through November 17. Survey respondents were recruited from the Understanding America Study (UAS), which is a household panel of the U.S. population maintained by the Center for Economic and Social Research at the University of Southern California.[37] The panel consists of approximately 2,000 individuals aged 18 and older, who have been randomly recruited using address-based sampling methods; participation in the UAS is voluntary, with individuals providing their written consent. The UAS is similar to the RAND American Life Panel, which was used to study (among other things) the value of insurance and health literacy.[38, 39]

UAS panelists are interviewed regularly over the Internet. An Internet survey may mitigate the tendency of respondents to answer questions the way they think a questioner might want, thereby minimizing social desirability bias.[40] While participating in repeated surveys could influence responses, there is no strong evidence of such an impact.[41, 42] UAS panelists typically have their own Internet access; those who do not are provided with a tablet and an Internet subscription.

In this survey, panelists received a nominal sum ($5) for their participation. A financial incentive has been found to improve response rates and, ultimately, representativeness and data quality.[43–46]

### Analysis

For each respondent, our survey elicited their willingness to use an Ebola vaccine under community vaccination rates of 10% and 90%. As discussed previously, willingness to use a vaccine could increase or decrease with the community vaccination rate. Thus, the null hypothesis which we tested is that stated willingness to use an Ebola vaccine did not vary with the community vaccination rate.

To test our hypothesis, we regressed these dichotomous responses from the survey questions on an indicator variable for a 90% rate. To understand the role of other factors, we then added out-of-pocket costs and respondent characteristics (e.g., age, gender and annual household income) to the regression; inclination to use the vaccine was then predicted for each respondent under the alternative community vaccination scenarios, and averaged across respondents.[47] Standard errors were clustered at the respondent level due to likely unobserved correlation across responses.

We explored the robustness of the results by replacing observed respondent characteristics with an indicator variable for each respondent to account for unobserved characteristics. In addition, we analyzed survey responses based on a logistic model instead of linear regression.

## Results

1,568 panelists in the Understanding America Study (UAS) were invited to participate in the survey, with 1,023 completing it (response rate of 65 percent) within the study window. Of these, 3 participants responded “don’t know / refuse” to the vaccination scenarios. Complete sociodemographic information was available for 1,003 of the remaining respondents. As Table 1 shows, respondents were identical to the U.S. population in terms of gender, but were somewhat older on average (48.5 years versus 47.0 years) and more likely to be non-Hispanic white, married, and currently working, and tended to have higher educational attainment and household income. At the time the survey was fielded (October/November 2014), 28% were concerned about getting sick from the disease. Across all survey scenarios (defined by community vaccination rates and out-of-pocket costs), 51% of responses indicated a willingness to use a potential Ebola vaccine.

**Table 1:** Summary Statistics from Ebola Survey (N = 1,003).

	Sample Mean	U.S. Average
Would purchase vaccine	51%	n/a
Concerned about Ebola	28%	n/a
Age	48.5 y	47.0 y
Female	51%	51%
Non-Hispanic white	72%	65%
Marital Status		
Never married	21%	28%
Married	56%	52%
Other	23%	20%
Currently working	62%	59%
Annual household income		
Below $20,000	17%	18%
$20,000–$50,000	28%	34%
$50,000–$100,000	34%	33%
Above $100,000	22%	15%
Educational attainment		
Less than high school	5%	12%
High School graduate	16%	30%
Some college	34%	29%
Bachelor's Degree	26%	19%
Graduate School	19%	10%

Notes: *n/a* is not available. See S1 Appendix Section A for information on U.S. averages.

**Fig 1:**
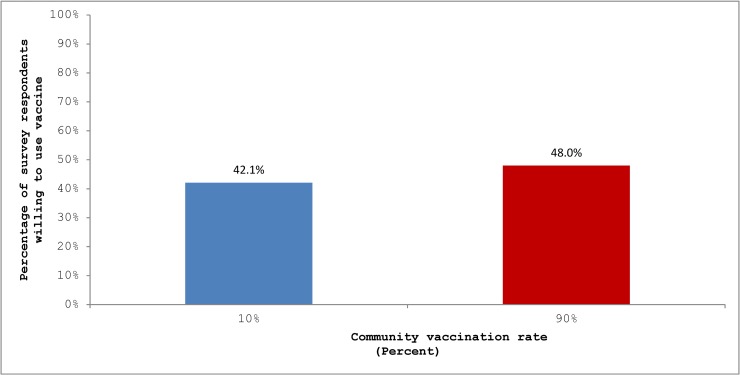
Individual Willingness To Use an Ebola Vaccine, by Community Vaccination Rate.

Survey respondents were presented with alternative scenarios about utilization of a vaccine within their communities. Fig 1 shows that a respondent’s stated willingness to use the vaccine increased from 42.1% to 48.0%—a relative increase of 14.0%—as the community vaccination rate increased from 10% to 90% (*p* value of less than 0.01 for the difference.) There was a nearly identical increase in the reported inclination to use a vaccine based on regressions that 1) included respondent characteristics as well as hypothetical out-of-pocket cost levels, 2) replaced observed respondent characteristics with indicator variables for each respondent to account for unobserved characteristics, and 3) used a logistic rather than linear model. Complete regression results are reported in S1 Appendix Section B.

**Fig 2:**
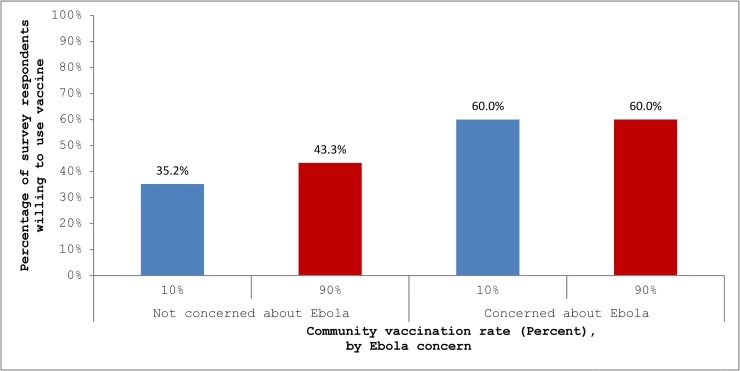
Individual Willingness To Use an Ebola Vaccine, by Community Vaccination Rate and Concern about Ebola.

We then assessed whether the response to community vaccination rates varied with whether an individual reported concern about getting sick from Ebola. To begin with, respondents who were concerned stated a greater willingness to use a vaccine, regardless of community vaccination rates (*p* < 0.01 in both cases.) For example, Fig 2 shows that 60.0% of concerned respondents would use the vaccine, compared to only 35.3% of respondents who were not concerned, if the community vaccination rate were 10%. For concerned respondents (28% of the total), reported inclination to use the vaccine was identical with 10% and 90% community vaccination rates. However, among respondents who were not concerned, stated willingness increased—from 35.3% to 43.3%, for a relative increase of 22.7%—with a community vaccination rate of 90% (*p* < 0.01.)

To put these findings in some context and to assess the face validity of our approach, we investigated the relationship between out-of-pocket costs and reported inclination to use the vaccine. Based on our primary regression, willingness to use a vaccine decreased from 61.0% to 40.4% as out-of-pocket cost increased from $25 to $100 (*p* < 0.01.) Thus, a decrease in the community vaccination rate from 90% to 10% has the same negative impact on individual willingness to use the vaccine as an increase in out-of-pocket cost from $25 to $47.

## Discussion

This study investigated the influence of peers on vaccination decision making by using a rapid survey technology to ask a large sample of U.S. adults in the fall of 2014 about their willingness to use a hypothetical vaccine for Ebola, calibrating its attributes to those of a vaccine which was then in development and has since completed a successful trial in west Africa.

Survey respondents were presented with two scenarios for vaccination rates within their community. At a low rate (10% of the community), 42.1% of respondents expressed a willingness to use the Ebola vaccine. For a 90% rate of community vaccination, the proportion of vaccine-willing respondents was significantly higher, reaching 48.0%. To put this finding into some perspective, this higher rate of community vaccination had the same impact on reported inclination to use the vaccine as a nearly 50% reduction in out-of-pocket cost.

It is possible that the relationship between an increase in the community vaccination rate and an individual’s willingness to use a vaccine varies with the level of the rate of vaccination in the community. Our study was not designed to address this hypothesis. It is nevertheless interesting to note that the reported willingness to use a vaccine was relatively high among individuals who were concerned about Ebola, and unrelated to the community rate. The incremental impacts of increased community vaccination must ultimately diminish, and the precise pattern merits further investigation.

Placing our findings in the context of the literature, Harmsen and colleagues surveyed parents of newborns and found that stated intentions to vaccinate against hepatitis B were significantly greater in relation to social norms supporting vaccination, defined by beliefs that most friends would vaccinate, that people important to the parents favored vaccination, and that these important people would appreciate the parents’ decision to vaccinate.[13] Hamilton-West as well as Rao and colleagues showed that college students were more likely to receive MMR and flu vaccines, respectively, when their peers were vaccinated.[14, 15]

These associations may not reflect a causal effect of peers on vaccination decisions. Individuals may self-select into communities based on shared values and behaviors.[48, 49] Moreover, in the cases of Hamilton-West and Rao and colleagues, it is difficult to demonstrate that a group’s behavior drives an individual’s decision making, when that individual is a member of the group and so helps determine the group’s behavior.[50] Our study avoided these challenges by asking each survey respondent how a hypothetical change in the community vaccination rate would affect the individual’s stated preference, with all other factors held constant.

Our study is limited by its reliance on “stated preferences,” rather than actual behavior. Survey results do not necessarily correspond to the real world, due to manipulated or merely inaccurate responses. Scholars have devoted considerable effort to developing valid stated-preference methods. In 1993, the National Oceanic and Atmospheric Association convened an expert panel, led by Nobel laureates, to consider how best to ascertain the societal value of goods—such as environmental preservation—which are not usually bought and sold in the marketplace.[34] The panel recommended the referendum format used here for its realism, and lack of incentive to misreport. Stated-preference methods have been applied to the context of vaccination [13, 22–25], and there is some evidence of substantial concordance with actual behavior.[51]

Beyond the reliance on stated preferences, our study is limited by the fact that our survey sample was not fully representative of U.S. adults. Furthermore, our results may not generalize to other populations or diseases.

Despite these limitations, the evidence here and elsewhere in the literature consistently points to positive peer effects in vaccination decision making, and so it is important to understand their nature. To begin with, the evidence does not necessarily imply that individuals do not “free ride” on the vaccination decisions of others. Increases in community vaccination rates reduce the risk of disease. If individuals respond to this reduced risk with less inclination to vaccination, then they are free riding on the vaccination decisions of others. In fact, the substantially greater willingness of survey respondents who were concerned about Ebola to use a vaccine evidences a linkage between perceived risk and inclination to vaccinate that is consistent with the herd immunity hypothesis. Our finding of a *higher* willingness to vaccinate when the community rate is high suggests that increases in community vaccination rate also have a positive spillover effect.

This evidence of positive effects could result from a desire to conform to the behavior of peers, or from learning about the favorable experiences of one’s peers. Rao and colleagues link vaccine use to learning about the health benefits. In our specific context, learning would seem to play a limited role. Participants in our survey were presented with information about treatment efficacy, side effects and mode of administration. Furthermore, a survey respondent would have had to anticipate that her beliefs about the vaccine were somehow pessimistic, and that her peers’ experiences would help her to develop a more accurate assessment.

Positive peer effects have significant implications for vaccination policy. A common view is that vaccination can be a victim of its own success. As more individuals are vaccinated, the benefits of further vaccination diminish. Under this view, vaccination within the population may level off at a rate below the level needed to control an outbreak of infectious disease. Financial subsidies can help to improve vaccination rates, but may have limited effectiveness.

Where individual vaccination decisions respond positively to community vaccination rates, a “multiplier effect” enhances the impact of programs. For example, free preventive care under the Affordable Care Act could encourage vaccination directly, by removing a financial barrier for some community members, but also indirectly, by strengthening norms which shape the decisions of others. As another example, peer effects could reinforce outreach efforts by the U.S. Centers for Diseases Control and Prevention and others to promote vaccination, including the recent use of social media campaigns, public service announcements, and online information clearinghouses. [2, 52, 53] The nature of positive peer effects in vaccination behavior, and the precise impacts on the effectiveness of vaccination programs, are important matters that need to be better understood.

## Supporting Information

S1 Appendix Section A. U.S. Averages in Table 1 Section B. Primary Regression Results Section C. Regression Results Underlying Fig 2. Section D. Complete Survey Instrument.
(PDF)

S1 File Data. (DTA)
