# Supplementary material for: National Survey Indicates that Individual Vaccination Decisions Respond Positively to Community Vaccination Rates
Source: PLoS One. 2016 Nov 21;11(11):e0166858. doi: 10.1371/journal.pone.0166858 (PMC5117726; doi:10.1371/journal.pone.0166858)
Supplement: S1 Appendix — Section A. U.S. Averages in Table 1 Section B. Primary Regression Results Section C. Regression Results Underlying Fig 2. Section D. Complete Survey Instrument. (PDF) [file pone.0166858.s001.pdf]

Section A: U.S. Averages in Table 1

Wherever possible, we obtained national averages for the adult population in 2014. We now describe our sources and, as needed, calculations for each population characteristic:

- *Age:* "Annual Estimates of the Resident Population by Single Year of Age and Sex for the United States and States: April 1, 2010 to July 1, 2014 – 2014 Population Estimates," produced by the U.S. Census Bureau. Note: To calculate average age among adults, residents 100 years old or older were treated as 100 years old.
- *Gender:* "Annual Estimates of the Resident Population by Sex, Age, Race, and Hispanic Origin for the United States and States: April 1, 2010 to July 1, 2014 – 2014 Population Estimates" from National Characteristics: Vintage 2014, produced by the U.S. Census Bureau.
- *Race / ethnicity:* Same as gender.
- *Marital status:* "Table A1. Marital Status Of People 15 Years And Over, By Age, Sex, Personal Earnings, Race, And Hispanic Origin: 2014" from America's Families and Living Arrangements: 2014: Adults (A table series), produced by the U.S. Census Bureau. Note: These data include 15-17 year olds.
- *Currently working:* "Employment-population ratio" from the Current Population Survey, produced by the Bureau of Labor Statistics. Note: These data include 16 and 17 year olds.
- *Household income:* "HINC-0, Selected Characteristics of Households by Total Money Income" from the Annual Social and Economic Supplement of the Current Population Survey, produced by the U.S. Census Bureau.
- *Educational attainment:* "Table 1. Educational Attainment of the Population 18 Years and Over, by Age, Sex, Race, and Hispanic Origin: 2014" from Educational Attainment in the United States: 2014 - Detailed Tables, produced by the U.S. Census Bureau.

Section B: Primary Regression Results

Specification	Unadjusted linear	Adjusted regression	Logistic model	Respondent fixed eff.
Coefficient[a] (Standard error)				
90 percent community vaccination rate (reference: 10 percent)	0.0588*** (0.0104)	0.0588*** (0.0105)	0.0588*** (0.0104)	0.0588*** (0.0104)
Out-of-pocket cost (reference: $25)				
$100	–	-0.205*** (0.0346)	-0.205*** (0.0344)	–
$250	–	-0.284*** (0.0341)	-0.283*** (0.0338)	–
Concerned about Ebola	–	0.208*** (0.0333)	0.208*** (0.0332)	–
Age	–	0.000146 (0.00118)	0.000160 (0.00117)	–
Female	–	0.0450 (0.0301)	0.0455 (0.0301)	–
Non-hispanic white	–	-0.0839** (0.0325)	-0.0841*** (0.0325)	–
Marital status (reference: single)				
Married	–	0.110*** (0.0407)	0.110*** (0.0404)	–
Separated	–	0.122*** (0.0472)	0.123*** (0.0472)	–
Currently working	–	-0.0218 (0.0350)	-0.0223 (0.0349)	–
Annual household income (reference: below $20,000)				
$20,000 - $50,000	–	0.0204 (0.0463)	0.0211 (0.0469)	–
$50,000 - $100,000	–	0.0307 (0.0497)	0.0317 (0.0503)	–
Above $100,000	–	0.0606 (0.0576)	0.0617 (0.0586)	–
Educational attainment (reference: less than high school)				
High school graduate	–	-0.0498 (0.0767)	-0.0492 (0.0737)	–
Some college	–	0.00379 (0.0738)	0.00435 (0.0719)	–
Bachelor's degree	–	0.0206 (0.0772)	0.0209 (0.0754)	–
Graduate school	–	0.0344 (0.0796)	0.0365 (0.0783)	–
Constant	0.421*** (0.0156)	0.441*** (0.0998)	–	0.421*** (0.00738)
Other statistics				
Observations	2,006	2,006	2,006	2,006
R-squared	0.003	0.112	0.085	0.890

[a]Notes: Dependent variable indicates willingness to use Ebola vaccine. Logistic model reports average marginal effects. Standard errors are clustered at the respondent level except for respondent fixed effects specification. ***, **, and * represent significance at the 1 percent, 5 percent and 10 percent levels, respectively.

Section C: Regression Results Underlying Figure 2

Specification	Adjusted Regression
Coefficient (Standard error)	
90 percent community vaccination rate (reference: 10 percent)	0.0813***
	(0.0122)
Concerned about Ebola	0.248***
	(0.0351)
90 percent community vaccination rate X Concern about Ebola	-0.0813***
	(0.0233)
Out-of-pocket cost (reference: $25)	
$100	-0.205***
	(0.0346)
$250	-0.284***
	(0.0341)
Age	0.000146
	(0.00118)
Female	0.0450
	(0.0301)
Non-hispanic white	-0.0839**
	(0.0326)
Marital status (reference: single)	
Married	0.110***
	(0.0407)
Separated	0.122***
	(0.0472)
Currently working	-0.0218
	(0.0350)
Annual household Income (reference: below $20,000)	
$20,000 - $50,000	0.0204
	(0.0463)
$50,000 - $100,000	0.0307
	(0.0497)
Above $100,000	0.0606
	(0.0576)
Educational attainment (reference: Less than high school)	
high school graduate	-0.0498
	(0.0767)
Some college	0.00379
	(0.0738)
Bachelor's degree	0.0206
	(0.0772)
Graduate school	0.0344
	(0.0796)
Constant	0.430***
	(0.0999)
Other statistics	
Observations	2,006
R-squared	0.113

Note: Specification corresponds to Column 2 of Appendix Table 1, with interaction added. tandard errors are clustered at the individual level. ***,**,* represent significance at the 1 percent, 5 percent and 10 percent levels, respectively.

Section D: Complete Survey Instrument

We'd now like to ask you for your thoughts on some issues related to the Ebola virus / disease.

How much, if anything, have you read or heard about the current outbreak of the Ebola virus / disease? Have you heard
1 A lot
2 A little
3 Nothing at all
4 Don't know / refused

[IF NOTHING AT ALL OR DON'T KNOW, PROCEED. OTHERWISE, SKIP TO SKIP POINT A.]

According to the World Health Organization, "Ebola virus disease" is a severe illness in humans.

The virus spreads via direct contact with the blood or other bodily fluids of infected people, and with materials contaminated with these fluids.

About 50% of people who are infected with the Ebola virus die. While treatment can improve survival, there is currently no cure for Ebola.

The first Ebola outbreaks happened in 1976 in Central Africa.

A new Ebola outbreak started earlier this year. As of October, 2014, nearly 10,000 people had been infected, almost all of them in West Africa. In the United States, four cases of Ebola had been diagnosed as of October 23.

There is currently no vaccine that prevents infection. Community engagement is key to successfully controlling outbreaks.

[SKIP TO SKIP POINT B]

[SKIP POINT A]

What are your sources of information about the Ebola virus? Select all that apply
1 Local newspaper
2 Other newspaper
3 Local television
4 Other television
5 Local radio
6 Other radio
7 Twitter
8 Other social media
9 Internet search engine
10 Other internet
11 My doctor
12 Public health officials
13 Elected leaders
14 Word of mouth from friends, family, co-workers or neighbors
15 Other

[INCLUDE BLANK TO BE FILLED WITH OTHER]

[SKIP POINT B]

How worried are you, if at all, that the U.S. will see a large number of Ebola cases in the next 12 months? Are you very worried, somewhat worried, not too worried, or not at all worried?
1 Very worried
2 Somewhat worried
3 Not too worried
4 Not at all worried

How worried are you, if at all, that you or someone in your family will get sick from Ebola? Are you very worried, somewhat worried, not too worried, or not at all worried?
1 Very worried
2 Somewhat worried
3 Not too worried
4 Not at all worried

How worried are you, if at all, that the <u>rest of the world</u> will see a large number of Ebola cases in the next 12 months? Are you very worried, somewhat worried, not too worried, or not at all worried?
1 Very worried
2 Somewhat worried
3 Not too worried
4 Not at all worried

4. What is your best guess of the number of people throughout the world who have died from the Ebola virus during the current outbreak?

____

What is your best guess of the number of people in the United States who die each year from the seasonal flu?

____

What is your best guess of the number of people in the world who die each year from the mosquito-borne disease malaria?

____

Do you think the U.S. government is doing too much, too little, or about the right amount to help develop a vaccine that would prevent people from getting the Ebola virus?
1 Too much
2 Too little
3 About the right amount
4 Don't know / refuse

What is your best guess of the amount of money the U.S. government spends in a typical year on scientific research about the Ebola virus?

____

What is your best guess of the amount of money the U.S. government spends in a typical year on scientific research about the seasonal flu?

____

What is your best guess of the amount of money the U.S. government spends in a typical year on scientific research about malaria?

____

A number of vaccines for Ebola are being developed.

It is possible that a vaccine will have these features:

- *You can take the vaccine by getting a shot in your arm*
- *The vaccine protects you from getting infected with the Ebola virus for fourteen (14) months*
- *There are no serious "side effects" for your health*
- *The cost is $RANDOMIZED*

In addition, suppose that one out of ten (10% of) people in your community are using the vaccine.

Would you pay the $RANDOMIZED cost out of your own money to take the vaccine yourself?
1 Yes
2 No
3 Don't know / refuse

Suppose that the cost of the vaccine is again RANDOMIZED.

Now, however, nine out of ten (90% of) people in your community are using the vaccine.

Everything else is the same as before.

Would you pay the $RANDOMIZED of your own money to take the vaccine?
1 Yes
2 No
3 Don't know / refuse

Again suppose that one out of ten people in your community are using the vaccine shots.

However, a second "booster" shot is required, and it keeps you from getting Ebola for ten (10) months.

The total cost of the two shots is $RANDOMIZED.

Would you pay the $ RANDOMIZED cost to take the shots?
1 Yes
2 No
3 Don't know / refuse

How did the recent cases of Ebola in the United States affect you, if at all? Select all that apply

1 I washed my hands more frequently, or wore a mask
2 I considered my plans in the event of a wider outbreak
3 I experienced anxiety or stress
4 I stayed home from work
5 I kept my child / children home from school
6 I avoided other public places <u>in my community</u>
7 I cut back on travel
8 Other

[INCLUDE BLANK TO BE FILLED WITH OTHER]

Suppose there was <u>another case</u> of Ebola in the <u>United States</u>. How would you be affected, if at all? Select all that apply
1 I would wash my hands more frequently, or wear a mask
2 I would consider an emergency / disaster plan
3 I would experience anxiety or stress
4 I would stay home from work
5 I would keep my child / children home from school
6 I would avoid other public places in my community
7 I would cut back on travel
8 Other

[INCLUDE BLANK TO BE FILLED WITH OTHER]

Suppose there was <u>one (1) case</u> of Ebola in <u>your community</u>. How would you be affected, if at all? Select all that apply
1 Wash my hands more frequently, or wear a mask
2 Consider an emergency / disaster plan
3 Experience anxiety or stress
4 Stay home from work
5 Keep my child / children home from school
6 Avoid other public places in my community
7 Cut back on travel
8 Other

[INCLUDE BLANK TO BE FILLED WITH OTHER]

Suppose there were a total of ten (10) new Ebola cases spread across ten (10) different states. How would you be affected, if at all? Select all that apply

1 Wash my hands more frequently, or wear a mask
2 Consider an emergency / disaster plan
3 Experience anxiety or stress
4 Stay home from work
5 Keep my child / children home from school
6 Avoid other public places in my community
7 Cut back on travel
8 Other ____________ (Please fill in the blank)

How worried are you, if at all, that some people will be treated unfairly due to concerns about Ebola? Are you very worried, somewhat worried, not too worried, or not at all worried?

1 Very worried
2 Somewhat worried
3 Not too worried
4 Not at all worried

[IF VERY OR SOMEWHAT WORRIED, ASK THE FOLLOWING]

Why and how do you worry that some people will be treated unfairly due to concerns about Ebola?

[INCLUDE BLANK TO BE FILLED]
